# Whole Exome Sequencing Identifies New Causative Mutations in Tunisian Families with Non-Syndromic Deafness

**DOI:** 10.1371/journal.pone.0099797

**Published:** 2014-06-13

**Authors:** Zied Riahi, Crystel Bonnet, Rim Zainine, Malek Louha, Yosra Bouyacoub, Nadia Laroussi, Mariem Chargui, Rym Kefi, Laurence Jonard, Imen Dorboz, Jean-Pierre Hardelin, Sihem Belhaj Salah, Jacqueline Levilliers, Dominique Weil, Kenneth McElreavey, Odile Tanguy Boespflug, Ghazi Besbes, Sonia Abdelhak, Christine Petit

**Affiliations:** 1 Laboratoire de Génomique Biomédicale et Oncogénétique, Institut Pasteur de Tunis, Tunis, Tunisia; 2 Faculté des Sciences de Tunis, Université de Tunis El Manar, Tunis, Tunisia; 3 INSERM UMRS 1120, Institut de la Vision, Paris, France; 4 Service d'ORL et de Chirurgie Maxillo-faciale, CHU La Rabta, Tunis, Tunisia; 5 Centre de Référence des Surdités Génétiques, Hôpital Trousseau- APHP, Paris, France; 6 INSERM U931, Clermont Ferrand, France; 7 Unité de Génétique et Physiologie de l'Audition, Institut Pasteur de Paris, Paris, France; 8 Unité de Génétique du Développement Humain, Institut Pasteur de Paris, Paris, France; University of Bonn, Institut of experimental hematology and transfusion medicine, Germany

## Abstract

Identification of the causative mutations in patients affected by autosomal recessive non syndromic deafness (DFNB forms), is demanding due to genetic heterogeneity. After the exclusion of *GJB2* mutations and other mutations previously reported in Tunisian deaf patients, we performed whole exome sequencing in patients affected with severe to profound deafness, from four unrelated consanguineous Tunisian families. Four biallelic non previously reported mutations were identified in three different genes: a nonsense mutation, c.208C>T (p.R70X), in *LRTOMT*, a missense mutation, c.5417T>C (p.L1806P), in *MYO15A* and two splice site mutations, c.7395+3G>A, and c.2260+2T>A, in *MYO15A* and *TMC1* respectively. We thereby provide evidence that whole exome sequencing is a powerful, cost-effective screening tool to identify mutations causing recessive deafness in consanguineous families.

## Introduction

Profound congenital deafness affects about 1 out of 1000 newborns [1], and is mainly of genetic origin. Non-syndromic (isolated) deafness accounts for approximately 70% of inherited cases. To date, around 70 genes and more than 1000 mutations causing non-syndromic deafness have been reported (http://deafnessvariationdatabase.org). More than 45 genes and 69 loci are associated with autosomal recessive non-syndromic deafness (DFNB). Despite the broad genetic heterogeneity of DFNB, loss-of-function mutations in a single gene, *GJB2* (encoding connexin-26) account for more than 30% of the cases in most populations around the Mediterranean sea [2]. After the exclusion of *GJB2* in DFNB patients, finding the gene implicated is difficult due to the high degree of genetic heterogeneity [3]. In addition, many deafness genes consist of long and/or numerous exons, making conventional methods of mutation screening very expensive and time-consuming [4]. Recent advances in DNA enrichment and next generation sequencing techniques, however, allow rapid and cost-effective analysis to identify the causative mutations in deaf patients [5]. To date, ten syndromic or non-syndromic deafness genes have been identified using targeted genomic enrichment and whole exome sequencing (WES): *TPRN, GPSM2, CEACAM16, SMPX, HSD17B4, HARS2, MASP1,OTOGL, DNMT1,* and *TSPEAR.* In addition several studies have shown the efficacy of WES to identify the causative mutations in recessive deafness forms [1], [4], [6], [7]. After the exclusion of *GJB2* mutations and of most mutations previously identified in deaf individuals from Tunisia, we carried out WES in six deaf patients from four unrelated consanguineous Tunisian families.

## Materials and Methods

### Patients

**Figure 1:**
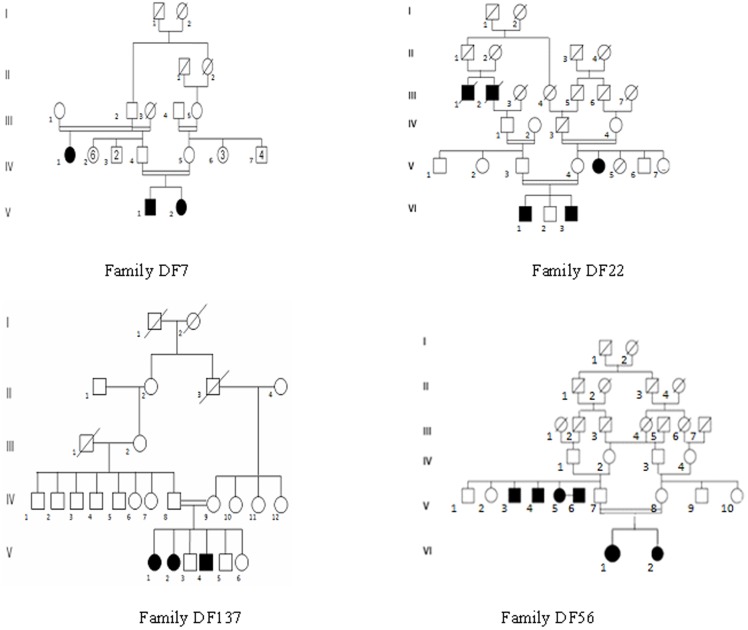
Pedigrees of the four Tunisian families analyzed using the whole exome sequencing strategy.

Four unrelated Tunisian families including deaf individuals were included in this study based on parental consanguinity and the presence of at least two affected siblings (**Figure 1**). Written informed consent was obtained from all participants or their legal guardians. Audiological evaluation was carried out at the Otorhinolaryngology Department at La Rabta hospital in Tunis. All patients had bilateral profound sensorineural deafness. Patients had the following clinical investigations: computed tomography of the temporal bones, auditory brainstem response, magnetic resonance imaging of the inner ear, tympanometry, fundus examination, cardiac and renal ultrasonography. Clinical examinations were unremarkable, and did not reveal symptoms or malformations that would suggest a syndromic form of deafness. Genomic DNA was extracted from peripheral blood samples using the standard salting-out method [8].

### Ethics statement

This study has obtained the ethics approval (IPT/LR11-05/Etude/05/2013) from the institutional review board of Pasteur Institute (Tunis- Tunisia- Registration number IRB00005445, FWA00010074). This study was conducted according to the principles of the declaration of Helsinki. Patients were anonymized and the corresponding code was conserved in a confidential file.

### Whole exome sequencing and bioinformatics analysis

A DNA pooling strategy was taken for family DF7 (patients V.1 and V.2) and for family DF56 (patients VI.1 and VI.2). For families DF22 and DF137, DNA was only available from one affected sibling in each family, i.e. VI.1 and V.1, respectively.

Targeted exome sequencing, library preparation, capture and sequencing, and sequence variant detection and annotation were performed by IntegraGen (Evry, France). Exons of genomic DNA samples were captured using the Agilent in-solution enrichment technique with a biotinylated oligonucleotide probe library, and paired-end 75-base massively parallel sequencing was carried out on an Illumina HiSeq2000. Sequence capture was performed according to the manufacturer's instructions (Human All Exon V5-50 Mb, Agilent). Briefly, 5 µg of each genomic DNA sample was fragmented by sonication and purified to yield fragments of 150–200 bp in length. Paired end adaptor oligonucleotides from Illumina were ligated on repaired A-tailed fragments that were purified and enriched by six polymerase chain reaction (PCR) cycles. Purified libraries (500 ng) were hybridized to the Sure Select oligonucleotide probe capture library for 24 h. After hybridization, washing and elution, the eluted fraction was PCR-amplified (10 to 12 PCR cycles), purified and quantified by quantitative PCR to obtain sufficient amounts of DNA template for downstream applications. Each eluted enriched DNA sample was then sequenced on an Illumina HiSeq 2000 as paired-end 75-base reads. Image analysis and base calling were performed using the Illumina Real-Time Analysis Pipeline version 1.14 with default parameters [9].

The exome design covers 51 Mb of the human genome corresponding to the exons and flanking intronic regions of 20 766 genes (220 000 exons) and also 700 miRNAs in the human reference sequence UCSC (hg19/GRCh37, February 2009 release) [7]. Bioinformatics analysis of sequencing data was based on the Illumina pipeline (CASAVA 1.8). CASAVA aligns reads to the human reference genome (hg19) with the alignment algorithm ELANDv2 (it performs multispeed and gapped alignments), calls SNPs on the basis of allele calls and read depth, and detects variants (SNPs and indels). Only positions included in the bait coordinates were conserved. Genetic variation was annotated with the IntegraGen in-house pipeline, consisting of gene annotation (using RefSeq), detection of known polymorphisms (using dbSNP132 and the 1000 Genomes Project database) and characterization of mutations as intronic or exonic, and silent, nonsense, missense and frame-shifting [9].

### Confirmation of the mutations by Sanger sequencing

Sanger sequencing was carried out to validate the mutations identified by WES as previously described [7]. Specific PCR and sequencing primers were designed using Primer3 (**Table 1**) (http://primer3.ut.ee/).

**Table 1:** Sequences of the primers used to validate the mutations by Sanger sequencing.

TMC1-EX22_23F	TTTAAGAAGTATCTTGGGGAACTG
TMC1-EX22_23R	ATGCCACTCACCATCCAATG
LRTOMT-7F	AGGATAATAATTGCTACTGGCAAAA
LRTOMT-7R	ATCCCAAATATTCCTTCACTGTCTT
MYO15A-EX21F	CCTTTTGCACATGGCTGAG
MYO15A-EX21R	GCCTGGGTTGTGTATTCCTG
MYO15A-EX36F	GGTGTGCTAGAATGGAGGCA
MYO15A-EX36R	GAGAGGTGGCAGTGGTGAC

## Results

Mutations in *GJB2*, the gene most frequently involved in autosomal recessive deafness in Tunisia [10]–[12], and mutations in other DFNB genes that had previously been reported in Tunisian deaf patients **(Table S1)** were first excluded by PCR and Sanger sequencing of these genes in the patients.

For the WES data analysis, based on familial history and pedigree we hypothesized an autosomal recessive mode of disease transmission and the presence of the causative mutations in the homozygous state in the patients.

To identify pathogenic variants, we filtered out polymorphisms using the Single Nucleotide Polymorphism Database dbSNP132. We excluded all the variants reported in 1000 genomes, Hapmap, and Exome variant server databases. In the second step, we focused on variants which are present in the coding exons and flanking splice sites. From the SNP and indels files, we selected nonsense, frame-shifting (indels), missense, and splice-site mutations, as they were more likely to be pathogenic. Only the variants with a read depth greater than 5 were retained.

After application of these 5 filtering steps (also listed in table 2), we found 3 SNPs and 1 indel inpatients DF7-V.1 and V.2, 11 SNPs and 1 indel inpatient DF22-VI.1, 3 SNPs and 1 indel inpatients DF56-VI.1 and VI.2, and 6 SNPs and 0 indel inpatient DF137-V.1 (**Table 2** **and Table S2**).

**Table 2:** Evolution of the number of variants during whole exome.

Patients	DF7-V.1 + V.2		DF22-VI.1		DF56-VI.1+VI.2		DF137-V.1	
Type of sequence variant	SNP	Indel	SNP	Indel	SNP	Indel	SNP	Indel
Total of variants	42959	6432	70417	5395	77906	6402	72500	5517
After exclusion of variants on chromosomes X and Y	42108	6234	67819	5275	73529	6210	70365	5357
After exclusion of heterozygous variants	12714	1635	28098	2162	21905	1733	26570	1901
After dbSNP132 filtering	247	374	266	601	114	450	179	470
After additional database filtering (Hapmap,1000G, Exome variant server)	32	17	92	30	26	47	68	22
after exclusion of intronic, 5′UTR, 3′UTR, and synonymous variants	3	1	11	1	3	1	6	0

The biallelic sequence variants predicted to be the causative mutations in the patients are all located in genes already known to be involved in deafness (*MYO15A*, *TMC1* and *LRTOMT*), but these particular mutations have not been previously reported (**Table 3**). Their presence in the homozygous state in all affected siblings, and in the heterozygous state in the clinically unaffected parents, was shown, in each family, by Sanger sequencing of the corresponding DNA fragment (except for the father in family DF56, whose DNA was not available). Finally, none of these mutations was present in the Exome Variant Server database or in 150 ethnically matched normally hearing individuals.

**Table 3:** Biallelic mutations identified in DFNB genes using a whole exome sequencing strategy.

Family	Genomic position(Hg19)	Gene	Refseq	Exon/intron	cDNA change	Amino acid change	Mutation type
DF7	chr.17 (18044343)	*MYO15A*	NM_01623	Exon 22	c.5417T>C	p.L1806P	missense
DF137	chr.17 (18054082)	*MYO15A*	NM_016239.3	Intron 37	c.7395+3G>A		splice site
DF22	Chr.11 (71817106)	*LRTOMT*	NM_001145309	Exon 7	c.208C>T	p.R70X	nonsense
DF56	chr.9 (75445600)	*TMC1*	NM_138691	Intron 23	c.2260+2T>A		splice site

## Discussion

We have identified previously unreported biallelic mutations in three different DFNB genes (*MYO15A*, *LRTOMT, TMC1*) in patients affected by congenital profound deafness, who belong to four unrelated Tunisian families, using a WES strategy.

In the DF7 patients, a missense mutation, c.5417T>C (p.L1806P), was identified in exon 22 of *MYO15A* (NM_016239). This mutation is predicted to be deleterious according to SIFT (http://sift.jcvi.org/) and Mutation Taster (http://www.mutationtaster.org/). In family DF137, a different mutation, c.7395+3G>A, was present in the same gene. This mutation is predicted to abolish the splice donor site of intron 37 and to create a cryptic splice donor site 3 bp upstream from the original site, according to the Alamut 2.3 software (http://www.interactive-biosoftware.com), which could lead to intron inclusion or exon skipping in the mature transcript. Mutations in *MYO15A* are responsible for the DFNB3 form of deafness [13]. The gene encodes myosin XVa, a 3530 amino acid motor protein involved in the differential elongation of the inner ear hair cells stereocilia [14], Two mutations in *MYO15A* had previously been identified in Tunisian patients whose hearing impairment loss ranged from severe to profound: c.7395+3G>C and c.4998C>A (p.C1666X) [15]. Mutations in *MYO15A* have also been identified in families originating from Pakistan, India [16] and Japan [17], and were associated with profound deafness in the patients. In family DF22, a nonsense mutation c.208C>T (p.R70X) was identified in exon 7 of *LRTOMT* (NM_001145309). The premature stop codon is predicted to result in a truncated protein with impaired function or no protein at all, due to nonsense mediated mRNA decay, according to Alamut 2.3 software. *LRTOMT* is responsible for the DFNB63 deafness form. It produces five different alternatively spliced transcripts, which encode two different leucine rich transmembrane and O- methyltransferase domain containing proteins, LRTOMT1 and LRTOMT2, both expressed in the inner ear sensory cells [18]. Mice carrying a mutation of the orthologous gene (*COMT2*) suffer from vestibular dysfunction, profound deafness and progressive degeneration of the organ of Corti [19]. Two mutations in *LRTOMT* had previously been reported in Tunisian patients, c.242G>A (p.R81Q) and c.313T>C (p.W105R). Two other mutations, c.358+4G>A and c.328G>A, have been identified in deaf patients from Pakistan and Turkey, respectively. In all cases, the hearing impairment was profound [18]. The p.R81Q mutation has also been found in deaf patients from Morocco at a frequency of 8.75%, which makes this gene the second most frequently involved deafness gene in Morocco, after *GJB2* [20]. Finally, the DF56 patients carried a c.2260+2T>A mutation in intron 23 of *TMC1* (NM_138691). According to Alamut 2.3, a cryptic splice donor site will be created 2 bp upstream from the original site, which is expected to result in the skipping of exon 23. *TMC1* has been implicated both in autosomal recessive (DFNB7/11) and autosomal dominant (DFNA36) deafness forms. In Tunisia, one particular mutation in exon 7 of *TMC1*, c.100C>T (p.R34X) has been identified in Tunisian patients affected by autosomal recessive non syndromic profound deafness at a frequency of 5.55% [21]. Mutations in this gene are also described in Turkey [22] and Pakistan [23] with prelingual severe to profound deafness. *TMC1* gene encodes a transmembrane channel-like protein and is expressed in mouse vestibular and cochlear hair cells. The precise role of the encoded protein remains to be established, but it has been to be required for mechanotransduction and proposed to be a component of the mechanotransduction channel. Its absence in TMC1 deficient mouse lead to hair cells degeneration [24].

In the present study, no genetic linkage analysis was performed prior to exome sequencing in order to identify candidate chromosomal regions and to reduce the analysis to those regions. Patients were selected based on familial history of deafness and the presence of parental consanguinity. Compared to the classical genetic method (homozygosity mapping and Sanger sequencing) WES is a cost effective strategy to identify mutations causing autosomal recessive deafness in consanguineous families.

Finally, since the Tunisian population shares a common genetic background with other populations of the Mediterranean basin and Middle- East [25], direct screening of the identified mutations in profoundly deaf patients from those regions would be useful to get an estimate of their prevalence.

## Supporting Information

Table S1 List of the mutations excluded by Sanger sequencing in ascertained families before whole exome sequencing.
(DOCX)

Table S2 List of the mutations retained after the 5 filtering steps.
(DOCX)
