# Supplementary material for: Whole Exome Sequencing Identifies New Causative Mutations in Tunisian Families with Non-Syndromic Deafness
Source: PLoS One. 2014 Jun 13;9(6):e99797. doi: 10.1371/journal.pone.0099797 (PMC4057390; doi:10.1371/journal.pone.0099797)
Supplement: Table S1 — List of the mutations excluded by Sanger sequencing in ascertained families before whole exome sequencing. (DOCX) [file pone.0099797.s001.docx]

| **Mutation** | **Gene** | **References** |
| --- | --- | --- |
| p.R34X | *TMC1* | [[1](#_ENREF_1)] |
| del [GJB6-D13S1830] and del [GJB6-D13S1854] | *GJB6* | [[2](#_ENREF_2)] |
| p.R81Q and p.W105R | *LRTOMT* | [[3](#_ENREF_3)] |
| p.Y305H | *ESRRB* | [[4](#_ENREF_4)] |
| p.W251C and p.P404L | *TMPRSS3* | [[5](#_ENREF_5)] |
| m.A1555G | *MT-RNR1* | [[6](#_ENREF_6),[7](#_ENREF_7)] |
| c.7395+3G>C and (p.C1666X) | *MYO15A* | [[8](#_ENREF_8)] |
| p.M599I | *MYO7A* | [[9](#_ENREF_9)] |

**Table S1**

1. Tlili A, Rebeh IB, Aifa-Hmani M, Dhouib H, Moalla J, et al. (2008) TMC1 but not TMC2 is responsible for autosomal recessive nonsyndromic hearing impairment in Tunisian families. Audiol Neurootol 13: 213-218.

2. Belguith H, Tlili A, Dhouib H, Ben Rebeh I, Lahmar I, et al. (2009) Mutation in gap and tight junctions in patients with non-syndromic hearing loss. Biochem Biophys Res Commun 385: 1-5.

3. Ahmed ZM, Masmoudi S, Kalay E, Belyantseva IA, Mosrati MA, et al. (2008) Mutations of LRTOMT, a fusion gene with alternative reading frames, cause nonsyndromic deafness in humans. Nat Genet 40: 1335-1340.

4. Ben Saïd M, Ayedi L, Mnejja M, Hakim B, Khalfallah A, et al. (2011) A novel missense mutation in the ESRRB gene causes DFNB35 hearing loss in a Tunisian family. European Journal of Medical Genetics 54: e535-e541.

5. Masmoudi S, Antonarakis SE, Schwede T, Ghorbel AM, Gratri M, et al. (2001) Novel missense mutations of TMPRSS3 in two consanguineous Tunisian families with non-syndromic autosomal recessive deafness. Hum Mutat 18: 101-108.

6. Mkaouar-Rebai E, Tlili A, Masmoudi S, Charfeddine I, Fakhfakh F (2008) New polymorphic mtDNA restriction site in the 12S rRNA gene detected in Tunisian patients with non-syndromic hearing loss. Biochem Biophys Res Commun 369: 849-852.

7. Mkaouar-Rebai E, Tlili A, Masmoudi S, Louhichi N, Charfeddine I, et al. (2006) Mutational analysis of the mitochondrial 12S rRNA and tRNASer(UCN) genes in Tunisian patients with nonsyndromic hearing loss. Biochem Biophys Res Commun 340: 1251-1258.

8. Belguith H, Aifa-Hmani M, Dhouib H, Said MB, Mosrati MA, et al. (2009) Screening of the DFNB3 locus: identification of three novel mutations of MYO15A associated with hearing loss and further suggestion for two distinctive genes on this locus. Genet Test Mol Biomarkers 13: 147-151.

9. Weil D, Kussel P, Blanchard S, Levy G, Levi-Acobas F, et al. (1997) The autosomal recessive isolated deafness, DFNB2, and the Usher 1B syndrome are allelic defects of the myosin-VIIA gene. Nat Genet 16: 191-193.
