# Supplementary material for: Whole Exome Sequencing Identifies New Causative Mutations in Tunisian Families with Non-Syndromic Deafness
Source: PLoS One. 2014 Jun 13;9(6):e99797. doi: 10.1371/journal.pone.0099797 (PMC4057390; doi:10.1371/journal.pone.0099797)
Supplement: Table S2 — List of the mutations retained after the 5 filtering steps. (DOCX) [file pone.0099797.s002.docx]

| Chromosome | Position | Gene | Refseq | Protein effect | Mutation type | Number of readings |
| --- | --- | --- | --- | --- | --- | --- |
| chr3 | 126707833 | *PLXNA1* | NM_03224 | p.A133T | missense | 179 |
| chr12 | 52842673 | *KRT6B* | NM_00555 | p.R386C | missense | 141 |
| chr17 | 18044343 | *MYO15A* | NM_01623 | p.L1806P | missense | 45 |
| chr17 | 27620990 | *NUFIP2* | NM_02077 | p.Gln29dup | duplication | 95 |

**Table S2**

1-DF7

2- DF22

| Chromosome | Position | Gene | Refseq | protein effect | Mutation type | Number of readings |
| --- | --- | --- | --- | --- | --- | --- |
| chr2 | 97779657 | *ANKRD36* | NM_00116431 | p.Arg61Glufs*42 | frame- shift | 14 |
| chr2 | 234171780 | *ATG16L1* | NM_03080 | p.G72R | missense | 146 |
| chr10 | 99024598 | *ARHGAP19* | NM_00125642 | p.E121K | missense | 59 |
| chr10 | 115910877 | *C10orf118* | NM_018017 | p.K288Q | missense | 100 |
| chr11 | 62381810 | *ROM1* | NM_00032 | p.P224L | missense | 72 |
| chr11 | 67074342 | *SSH3* | NM_01785 | p.L125F | missense | 24 |
| chr11 | 67209935 | *CORO1B* | NM_00101807 | p.S55R | missense | 22 |
| chr11 | 71817106 | *LRTOMT* | NM_00114530 | p.R70* | nonsense | 17 |
| chr12 | 29449986 | *FAR2* | NM_01809 | p.R133Q | missense | 43 |
| chr16 | 21098218 | *DNAH3* | NM_01753 | p.I943M | missense | 68 |
| chr17 | 56403014 | *BZRAP1* | NM_00475 | p.R211C | missense | 32 |
| chr19 | 1003331 | *GRIN3B* | NM_13869 | p.T210M | missense | 73 |

3-DF56

| Chromosome | Position | Gene | Refseq | cDNA / protein effect | Mutation type | Number of readings |
| --- | --- | --- | --- | --- | --- | --- |
| chr9 | 75445600 | *TMC1* | NM_138691 | c.2260+2T>A | splice site (intron) | 90 |
| chr10 | 74714326 | *PLA2G12B* | NM_032562 | p.R40W | missense | 194 |
| chr12 | 82792855 | *METTL25* | NM_032230 | p.K271N | missense | 179 |
| chr11 | 71498623 | *FAM86C1* | NM_001099653 | p.Ser15Phefs*30 | frame- shift | 10 |

4-DF137

| Chromosome | Position | Gene | Refseq | cDNA / protein effect | Mutation type | Number of readings |
| --- | --- | --- | --- | --- | --- | --- |
| chr1 | 151341655 | *SELENBP1* | NM_00394 | p.R62Q | missense | 44 |
| chr1 | 152281027 | *FLG* | NM_00201 | p.G2112E | missense | 65 |
| chr1 | 152323087 | *FLG2* | NM_00101434 | p.*2392S | missense | 89 |
| chr7 | 4827858 | *AP5Z1* | NM_01485 | p.R510W | missense | 22 |
| chr10 | 101578548 | *ABCC2* | NM_00039 | p.G758V | splice site (exon) | 27 |
| chr17 | 18054082 | *MYO15A* | NM_01623 | c.7395+3G>A | splice site (intron) | 17 |
